# Supplementary material for: Drosophila Kruppel homolog 1 represses lipolysis through interaction with dFOXO
Source: Sci Rep. 2017 Nov 27;7:16369. doi: 10.1038/s41598-017-16638-1 (PMC5703730; doi:10.1038/s41598-017-16638-1)
Supplement: Supplementary file 1 — Supplementary Information [file 41598_2017_16638_MOESM1_ESM.pdf]

Supplementary Information

Drosophila Kruppel homolog 1 represses lipolysis through interaction with dFOXO

Ping Kang [1], Kai Chang [1], Ying Liu [1], Mark Bouska [1], Allison Birnbaum [1], Galina Karashchuk [2], Rachel Thakore [2], Wenjing Zheng [2], Stephanie Post [2], Colin S. Brent [3], Sheng Li [4], Marc Tatar [2*], Hua Bai [1, 2*]

Supplementary Table S1. Putative KLF binding sites in the promoters of InR and bmm.

Supplementary Table S2. List of Primers

Supplementary figure S1. Original full-length blots for Figure 1C & 1D

Supplementary figure S2. Original full-length blots for Figure 2A

Supplementary figure S3. Original full-length blots for Figure 3A

Supplementary figure S4. Original full-length blots for Figure 3B

Supplementary figure S5. Original full-length blots for Figure 3C

Supplementary figure S6. Immunostaining to show the cytoplasmic expression of Kr-h1 in Kc167 cells

Supplementary Table S1. Putative KLF binding sites in the promoters of InR and bmm.

Brummer promoter						
**Model ID**	**Model name**	**Score**	**Relative score**	**Predicted site sequence**	**Start**	**End**
MA0599.1	KLF5	10.999	0.943175564	cccactccca	14778617	14778626
MA0599.1	KLF5	8.222	0.907848237	ctcactccca	14778639	14778648
MA0493.1	Klf1	12.451	0.922097346	agacccaccca	14778983	14778993
MA0039.2	Klf4	11.870	0.938178771	tgggtgggtc	14778984	14778993
MA0039.2	Klf4	11.760	0.936309891	ggggagtggc	14774266	14774275
MA0599.1	KLF5	13.400	0.973719645	gccactcccc	14774266	14774275
MA0741.1	KLF16	13.178	0.933335628	cacacaccccc	14774348	14774358
**InR promoter**						
**Model ID**	**Model name**	**Score**	**Relative score**	**Predicted site sequence**	**Start**	**End**
MA0599.1	KLF5	10.517	0.93704385	gctactcccc	17428300	17428309
MA0493.1	Klf1	11.370	0.903823178	agcccctccca	17429438	17429448
MA0039.2	Klf4	12.632	0.951125009	tgggaggggc	17429439	17429448
MA0741.1	KLF16	15.852	0.977047928	gccacgcccac	17441804	17441814
MA0493.1	Klf1	14.076	0.949567765	tgccacgccca	17441805	17441815
MA0039.2	Klf4	15.263	0.99582521	tgggcgtggc	17441805	17441814
MA0599.1	KLF5	13.501	0.975004507	gccacgccca	17441805	17441814
MA0599.1	KLF5	10.638	0.938583139	ttcccgccca	17442990	17442999
MA0493.1	Klf1	11.163	0.900323869	agatacaccca	17443145	17443155
MA0599.1	KLF5	8.711	0.914069002	tccccgccta	17445459	17445468

Supplementary Table S2. List of Primers

**Name**	**Directions**	**Sequence 5'->3'**	**Assays**
Kr-h1	Forward	TCA CAC ATC AAG AAG CCA ACT	qRT-PCR
	Reverse	GCTGGT TGG CGG AAT AGT AA	
bmm	Forward	GGCTACAATCATGTGCTGAAAC	qRT-PCR
	Reverse	CCTCCATCTCCTTCTTGTTCTT	
Lsd-1	Forward	CAAGGAGTACATGTCCGATCAC	qRT-PCR
	Reverse	CGGCTGCATAAGTGGTAAGT	
FASN1	Forward	TTCCACCGTGCCACTATTC	qRT-PCR
	Reverse	AGCTACCGCTCTCACAAATC	
dilp2	Forward	TCATCTCGATGGTGGCCGTGATTT	qRT-PCR
	Reverse	ACACCATACTCAGCACCTCGTTGA	
dilp5	Forward	GGTTGCCTGTCCCAATGGATTCAA	qRT-PCR
	Reverse	TATCCAAATCCGCCAAGTGGTCCT	
InR	Forward	TAT CCA AGA GTC CCG CAA AG	qRT-PCR
	Reverse	GGT CGT CGC TGT TAGTGG AG	
4ebp	Forward	CCATGATCACCAGGAAGGTTGTCA	qRT-PCR
	Reverse	AGCCCGCTCGTAGATAAGTTTGGT	
Act5C	Forward	TCGCGATTTGACCGACTACCTGAT	ChIP-PCR
	Reverse	TGATGTCACGGACGATTTCACGCT	
InR-P1	Forward	TGTGTGTGTGTATGTGTGTGTA	ChIP-PCR
	Reverse	TACAAGTGCGGGCGATTC	
InR-P2	Forward	TCTCCATTCCTGGTCCCATTA	ChIP-PCR
	Reverse	CTGCTTGGCCTTGAACTTAGA	
InR-P3	Forward	CAACTCGAACTTGCAACAAAGTA	ChIP-PCR
	Reverse	GTGGATTAAAGTGGCACGAATG	
bmm-P1	Forward	CACCGCGCCGCAATGAATGTATAA	ChIP-PCR
	Reverse	TTCAATCACTGTTTGTCGGTCGGC	
bmm-P2	Forward	AAGGAGCTGCAACGACTAAA	ChIP-PCR
	Reverse	CTTTGGACTCGGCGTTAGAT	
bmm-P3	Forward	AAATTGCAGCTGCCACAGTTCGTG	ChIP-PCR
	Reverse	TGCCATGAATTCTCCTCACTTGGC	
bmm-3'-UTR	Forward	AAACATCAGCGCCACAATTCTCCC	ChIP-PCR
	Reverse	ATATACATGTCGCTGCTGTGCGTG	

Supplementary figure S1. Original full-length blots for Figure 1C & 1D

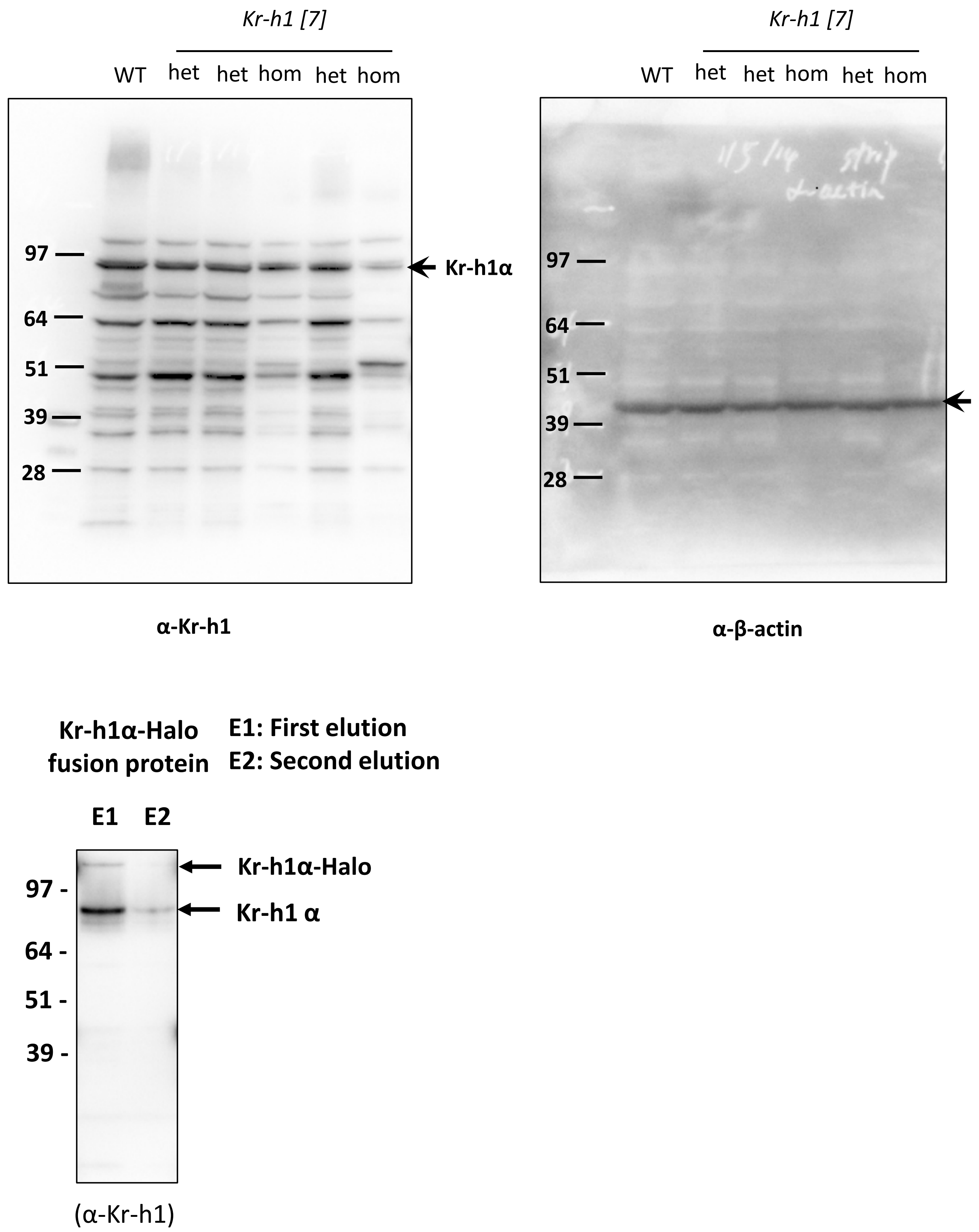

Supplementary figure S2. Original full-length blots for Figure 2A

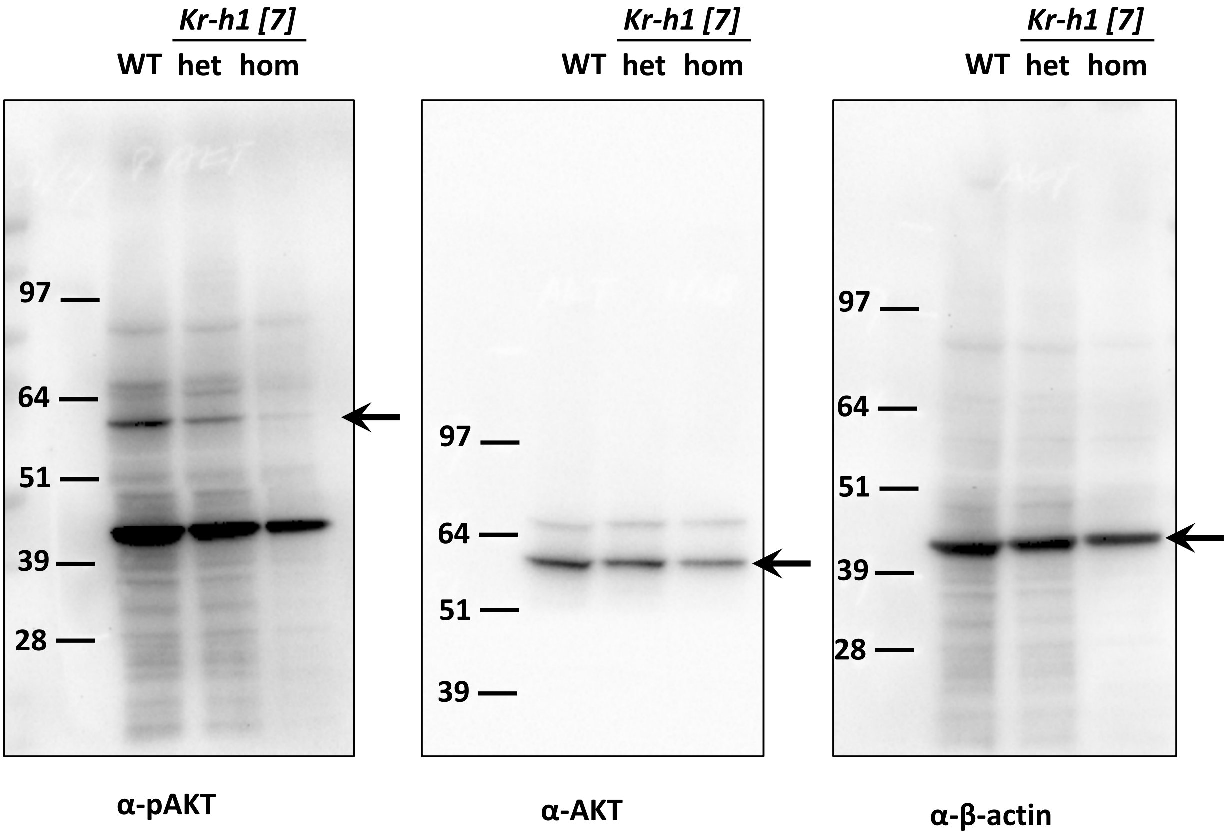

Supplementary figure S3. Original full-length blots for Figure 3A

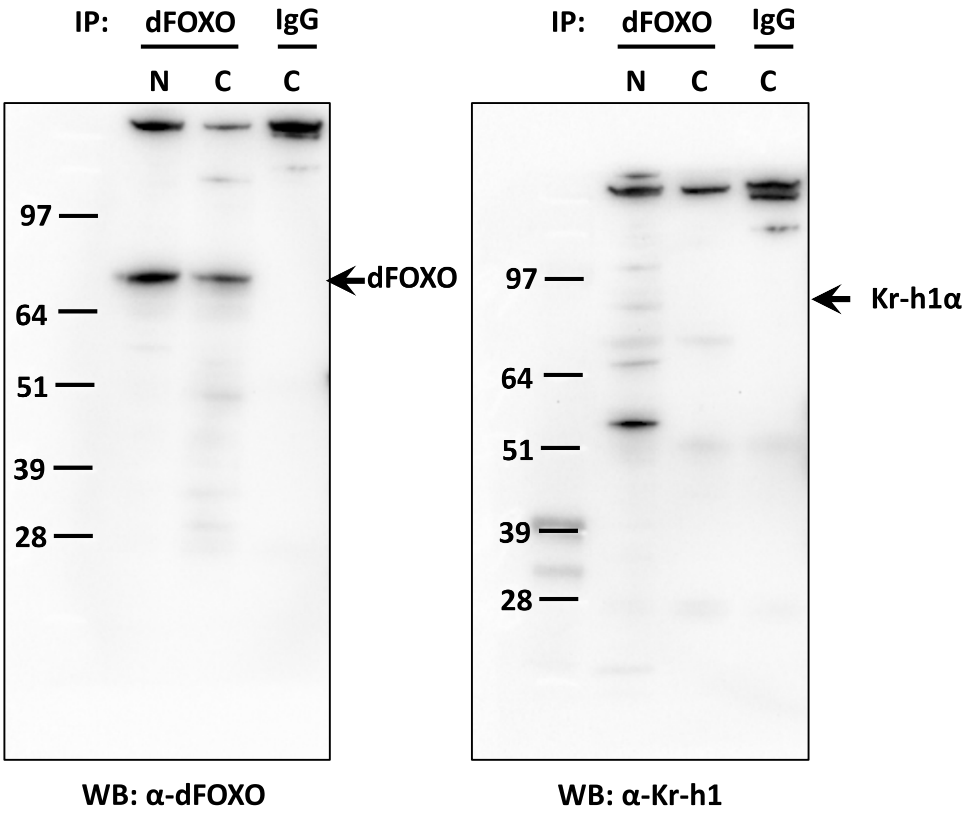

Supplementary figure S4. Original full-length blots for Figure 3B

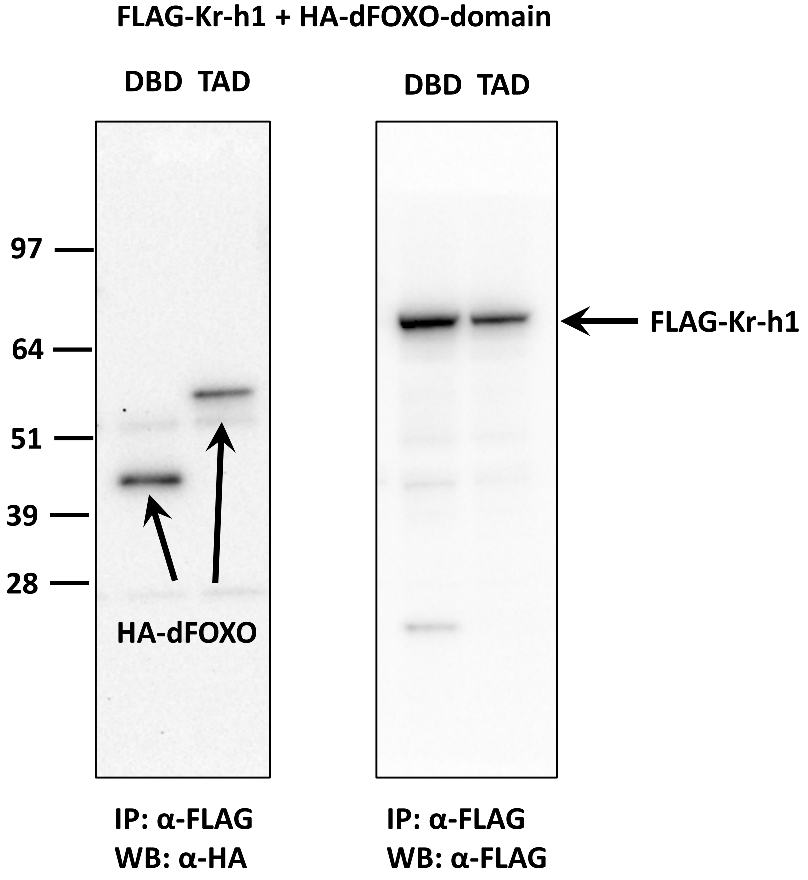

Supplementary figure S5. Original full-length blots for Figure 3C

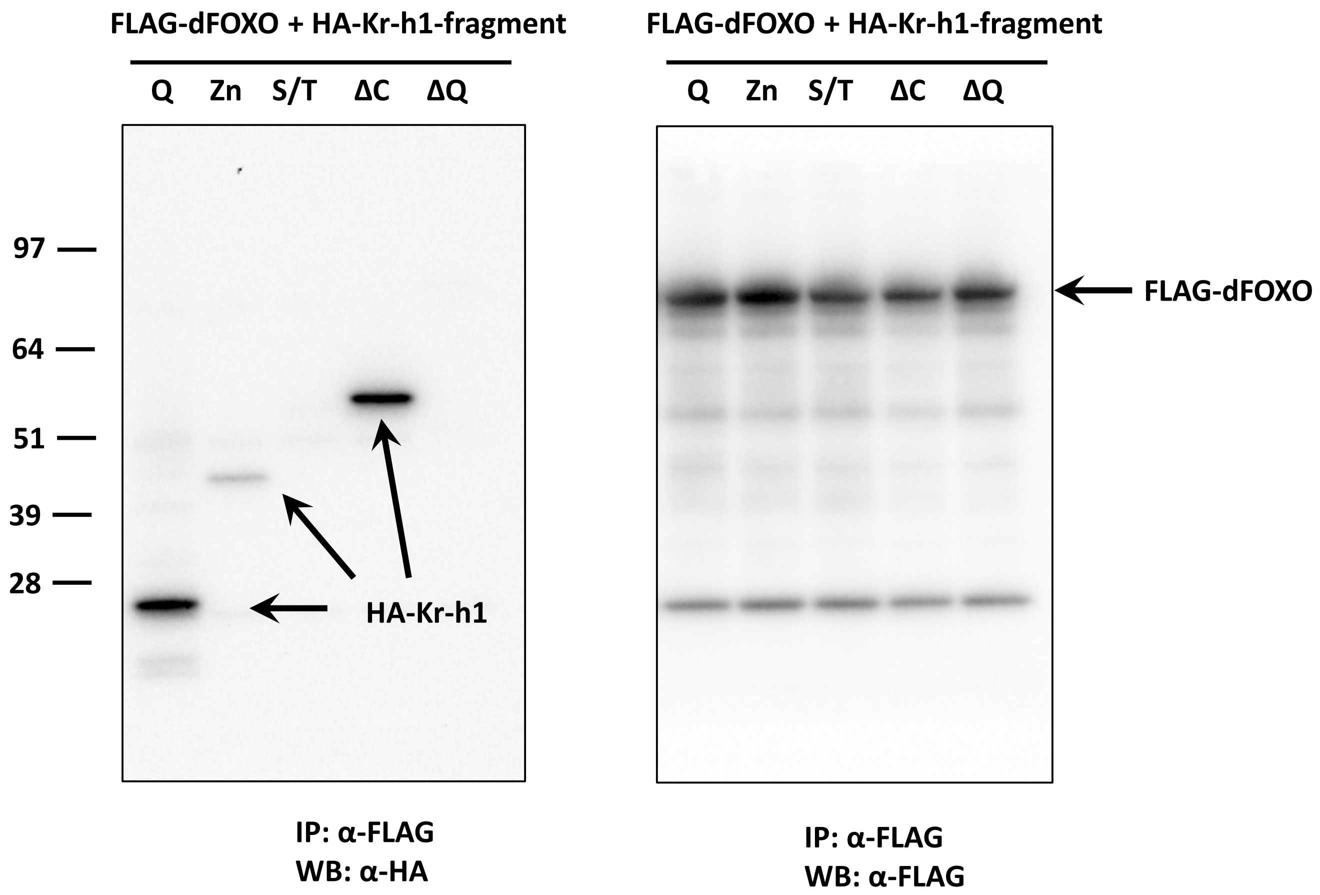

Supplementary figure S6. Immunostaining to show the cytoplasmic expression of Kr-h1 in Kc167 cells

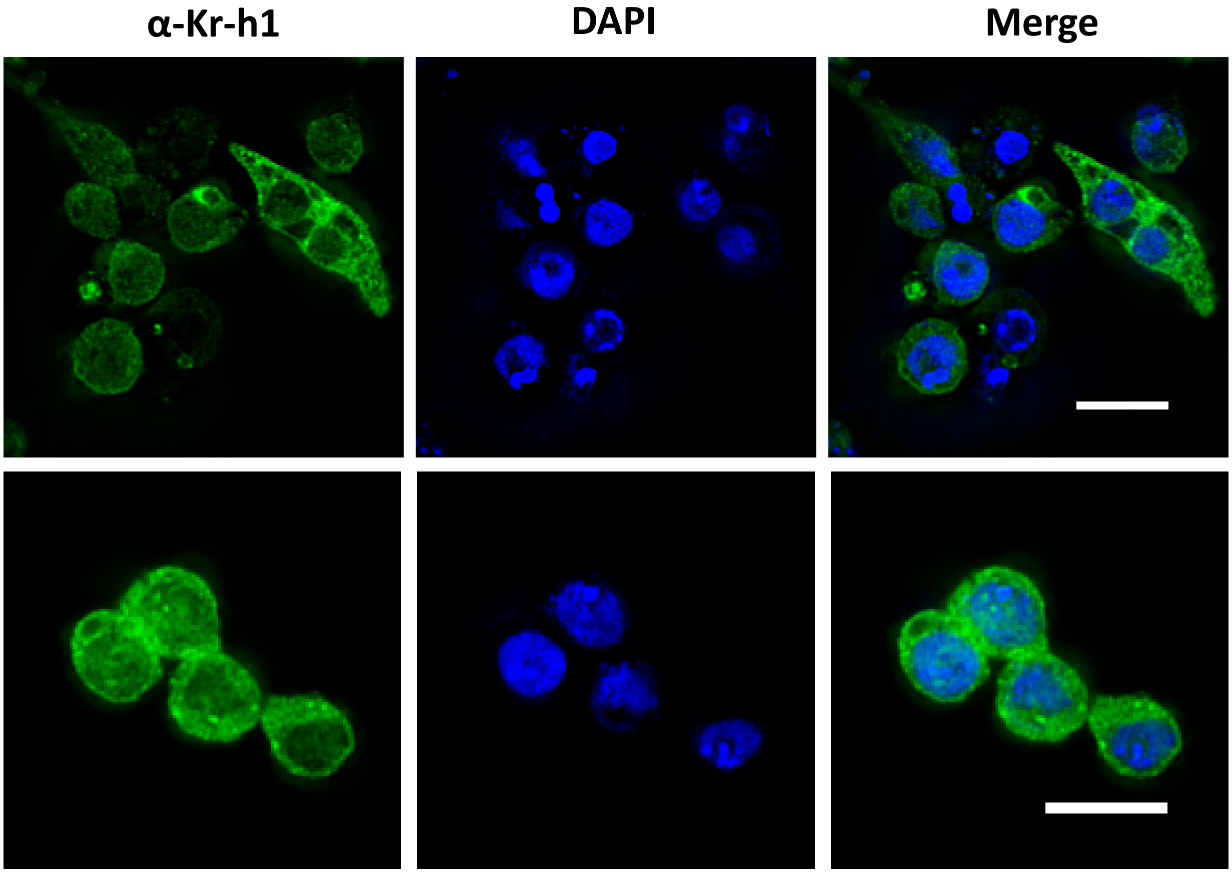

(Scale bar: 10 μm)
